# Practical Notes on Diagnosis and Treatment

**Published:** 1907-11-09

**Authors:** 


### Practical Notes on Diagnosis and Treatment.

**Asthma Powder.**

A well-known "asthma cure" is said to be composed as follows: "Powdered lobelia, black tea, and stramonium leaves, of each 1 ounce. Pour upon the mixture 2 ounces of a saturated solution of potassium nitrate and dry."

**Pleural Effusions.**

In chronic apyretic pleural effusions sodium salicylate sometimes acts well. It should be given in a cachet, and each dose is to be followed by a draught of some alkaline water. After three or four days the drug should be interrupted for a day or two, and then renewed.

**Glaucoma.**

The appearance of rings of colour around a flame is a frequent symptom in glaucoma. It always demands careful examination of the ocular tension. In glaucoma this is raised, the pupil is contracted, and cupping of the optic disc with pulsation of the retinal arteries may be noted.

**Ammonium Acetate as a Diaphoretic.**

The solution of ammonium acetate is largely prescribed as a diaphoretic in various febrile conditions, but very often too small a dose is ordered. Less than two drachms does not act on the skin, and three as an initial dose rapidly increased to six is a more appropriate quantity.

**Quinine in Uterine Inertia.**

Quinine is of great value in simple uterine inertia and to stimulate flagging pains in a primipara. Though less valuable than ergot in post-partum hæmorrhage, it is more serviceable than that drug in hæmorrhages occurring during labour. It should be given in a dose of 8 grains, this being followed, if necessary, by a dose of 4 grains, repeated on two occasions with an hour's interval.—*Dr. Owen C. Mackness.*

**Epilepsy.**

Professor Anthony Roche advocates strontium bromide in the treatment of epilepsy. He orders half a drachm in a bitter infusion night and morning, and if this proves inefficient he rapidly increases the dose. He has given as much as three drachms daily for weeks without unpleasant symptoms. In addition, he orders thirty grains to be given immediately when there is any warning of an attack. Failure in the use of this salt, Dr. Roche says, is due to insufficient dosage.

**Chloride of Sodium in Ringworm.**

Dr. Geo. Steele Perkins claims that the following method will almost invariably cure ringworm of the scalp, even in obstinate cases of long standing. Mix finely powdered sodium chloride with a little vaseline so as to make an ointment. Rub this into the affected area (previously shaved) night and morning until the place is sore—that is usually in three to four days. Then apply some simple application to promote healing. When this is secured, the parasite will be found to be absent, and healthy hairs will soon appear.

**Rheumatic Tonsillitis.**

Tonsillitis in children and young adults is frequently a sign of rheumatism. Hence complete rest should be prescribed and a careful watch should be kept on the heart.

**Tubercle and Pleurisy.**

The larger number of serous pleurisies are tuberculous, and after the disappearance or removal of the fluid from the chest, the subject of the disease remains tuberculous and ready to suffer from pulmonary invasion.—*Dr. Mitchell Bruce.*

**Renal Pain.**

When a stone is in the pelvis of the kidney, the pain shoots down to the inner side of the thigh and down to the testicle or labia, as the case may be. When the stone is in the kidney itself, there is a constant boring pain in the renal region.—*Dr. Hale White.*

**Movable Kidney.**

A large number of so-called movable kidneys should not be operated on at all, because the patient is merely neurotic. But a small number of cases require operating upon, because the effects are a serious disablement in life.—*Mr. Tubby.*

**The Pupils in General Paralysis.**

The condition of the pupils may vary in the same individual at different times. There may be irregularity of outline, inequality, contraction, or dilatation of pupils at one time and not at another. Even the Argyll-Robertson phenomena may be present, perhaps for weeks, and yet later the reaction to light may be normal.—*Dr. Jno. Macpherson.*

**Local Applications in Rheumatism.**

The following is recommended in acute rheumatism: Salicylic acid, oil of turpentine, lanoline, of each $\frac{1}{2}$ ounce, lard to 3 ounces. Apply to the painful joints on lint, and cover with gutta-percha tissue and a flannel bandage. In chronic rheumatism and gout the following may be tried: Salicylic acid, 300 grains; rectified spirit, 3 ounces; castor oil, 6 ounces. Lint soaked in this is applied as already described.

**Hæmoglobinuria.**

In addition to the paroxysmal form of this condition, which, by the way, is sometimes associated with Raynaud's phenomenon, it is well to remember that hæmoglobinuria occurs in congenital syphilis, and it has also been recorded in the acquired form of specific disease. Chlorate of potassium in excessive doses is another agent which sometimes causes hæmoglobinuria.

**Atropine in Asthma.**

Von Noorden confirms Trousseau's recommendation of atropine in the treatment of asthma. Beginning with $\frac{1}{150}$ grain the dose is increased to $\frac{1}{65}$ grain in two or three days, and then gradually to $\frac{1}{16}$ grain. Then the dose is gradually reduced, the whole course lasting from four to six weeks. The effect is to lengthen the interval between the attacks rather than to diminish the severity of the individual seizures.

